# Human cytomegalovirus uracil DNA glycosylase associates with ppUL44 and accelerates the accumulation of viral DNA

**DOI:** 10.1186/1743-422X-2-55

**Published:** 2005-07-15

**Authors:** Mark N Prichard, Heather Lawlor, Gregory M Duke, Chengjun Mo, Zhaoti Wang, Melissa Dixon, George Kemble, Earl R Kern

**Affiliations:** 1 Department of Pediatrics, University of Alabama at Birmingham, Birmingham AL, USA; 2 Department of Research, MedImmune Vaccines Inc., Mountain View, CA, USA

## Abstract

**Background:**

Human cytomegalovirus *UL114 *encodes a uracil-DNA glycosylase homolog that is highly conserved in all characterized herpesviruses that infect mammals. Previous studies demonstrated that the deletion of this nonessential gene delays significantly the onset of viral DNA synthesis and results in a prolonged replication cycle. The gene product, pUL114, also appears to be important in late phase DNA synthesis presumably by introducing single stranded breaks.

**Results:**

A series of experiments was performed to formally assign the observed phenotype to pUL114 and to characterize the function of the protein in viral replication. A cell line expressing pUL114 complemented the observed phenotype of a *UL114 *deletion virus in *trans*, confirming that the observed defects were the result of a deficiency in this gene product. Stocks of recombinant viruses without elevated levels of uracil were produced in the complementing cells; however they retained the phenotype of poor growth in normal fibroblasts suggesting that poor replication was unrelated to uracil content of input genomes. Recombinant viruses expressing epitope tagged versions of this gene demonstrated that pUL114 was expressed at early times and that it localized to viral replication compartments. This protein also coprecipitated with the DNA polymerase processivity factor, ppUL44 suggesting that these proteins associate in infected cells. This apparent interaction did not appear to require other viral proteins since ppUL44 could recruit pUL114 to the nucleus in uninfected cells. An analysis of DNA replication kinetics revealed that the initial rate of DNA synthesis and the accumulation of progeny viral genomes were significantly reduced compared to the parent virus.

**Conclusion:**

These data suggest that pUL114 associates with ppUL44 and that it functions as part of the viral DNA replication complex to increase the efficiency of both early and late phase viral DNA synthesis.

## Background

The enzymatic removal of uracil from DNA occurs in all free-living organisms. Both the misincorporation of dUTP by DNA polymerase and the spontaneous deamination of cytosine are relatively frequent events and give rise to uracil residues covalently linked to the genome, with the latter resolving into A:T transition mutations in one of the nascent strands [4,42]. Human herpesviruses, poxviruses and retroviruses either encode or recruit uracil DNA glycosylase (UNG) homologs, presumably to remove uracil bases from genomic DNA [5]. A number of studies used site directed mutagenesis to characterize the function of this gene in the life cycle of these viruses and most have described unexpected facets of the phenotype that involve DNA (or RNA) replication [5]. Studies described here with human cytomegalovirus (CMV) suggest that the UNG is part of the replication complex and that it functions in the replication of the viral genome.

Highly conserved mechanisms have evolved to minimize the presence of uracil in genomic DNA, presumably to prevent damage to the genome [30,44,46]. In humans, at least five base excision repair enzymes are capable of removing uracil bases incorporated in DNA. The human *UNG *gene expresses distinct nuclear and mitochondrial forms of this enzyme, designated UNG2 and UNG1, respectively [18]. In addition, a thymine(uracil) DNA glycosylase, a cyclin-like UNG, and a new gene SMUG1 have all been shown to possess this activity [24,26,27]. The relative function of each of these molecules remains to be characterized, but it appears that these molecules have developed specialized roles in mammals. Recent studies describing the phenotype of UNG knockout mice did not identify a greatly increased spontaneous mutation rate, in contrast to studies in both prokaryotes and *sacharomyces *[18]. SMUG1 appears to be responsible for recognizing and repairing uracil residues resulting from the spontaneous deamination of cytosine [26], whereas UNG2 colocalizes with replication foci in dividing cells and is thought to remove uracil during the replication process [18]. An ancillary role for this enzyme in mammalian DNA replication is also supported by the fact that UNG2 interacts physically with both replication protein A [25], as well as proliferating cell nuclear antigen (PCNA) which is a central regulator of DNA synthesis [28]. Further, these interactions suggest that UNG2 participates in the PCNA-requiring 2–8 bp patch base excision repair pathway [39].

A number of virus families appear to recruit UNG2, or to encode UNG2 homologs for use in the replication process. In human immunodeficiency virus (HIV) type 1, the vpr gene product interacts specifically with UNG2 [3]. The Vpr from simian immunodeficiency virus also binds UNG2 in a similar manner, however, it doesn't appear to impact the phenotype of cell cycle arrest associated with Vpr [38]. UNG2 is packaged inside retrovirus virions by an integrase dependent mechanism [45], and physically associates with integrase as well as reverse transcriptase in the pre-integration complex [33]. Lysates from purified virions demonstrated that UNG2 remained functional and was capable of directing the repair of uracil from a synthetic oligonucleotide template in conjunction with reverse transcriptase in a manner that is independent of apurinic/apyrimidinic endonuclease [33]. The function that UNG2 serves in HIV replication is unclear. However, the misincorporation of dUTP in a RT/RNAse H assay does not appear to affect first strand DNA synthesis by RT, but rather, it affects the specificity of cleavage by RNAse H resulting in reduced second strand synthesis from the RNA primers [17]. Poxviruses also encode a UNG2 homologs that perform an essential function in the replication of this virus [22,41,43] and are thought to act at the level of DNA synthesis [8]. More recent studies confirmed that D4R is essential for vaccinia DNA synthesis, and that its essential function is unrelated to its ability to excise uracil from DNA [7].

Herpesviruses all encode UNG homologs that do not appear to be required for replication in cell culture [23,31,36], although the deletion of the homolog in herpes simplex virus appears to reduce neuroinvasiveness in animal models [35]. CMV is unique among these viruses in that the deletion of this ORF results in a distinct phenotype characterized by a marked delay in the onset of DNA synthesis despite the normal temporal expression of early genes involved in this process [29,31]. The phenotype is less apparent in rapidly dividing cells, suggesting that a cellular gene might compensate at least to some degree [6]. Another interesting aspect of the UNG^- ^phenotype occurs late in infection where the mutant virus fails to initiate robust DNA synthesis and concurrently fails to incorporate uracil in the genome, suggesting that the removal of these moieties may be related to the switch to late phase DNA synthesis [6]. It is unclear why this phenotype is observed in CMV and not in other herpesviruses, but it may be related to the distinct mechanisms that this virus has evolved to replicate its genome that is independent of origin binding proteins encoded by most other herpesviruses.

To help understand how the *UL114 *gene product functions in viral DNA synthesis, a complementing cell line was constructed and recombinant viruses in which this gene product was epitope tagged were used to characterize its expression and localization in the context of a viral infection. Herein, we demonstrate that pUL114 localizes to the viral replication compartments and associates with the accessory factor of the DNA polymerase (ppUL44, ICP36), and that the absence of this molecule results in delayed onset of viral DNA synthesis as well as inefficient replication of the viral genome.

## Results

### Restoration of *UL114*

**Figure 1:**
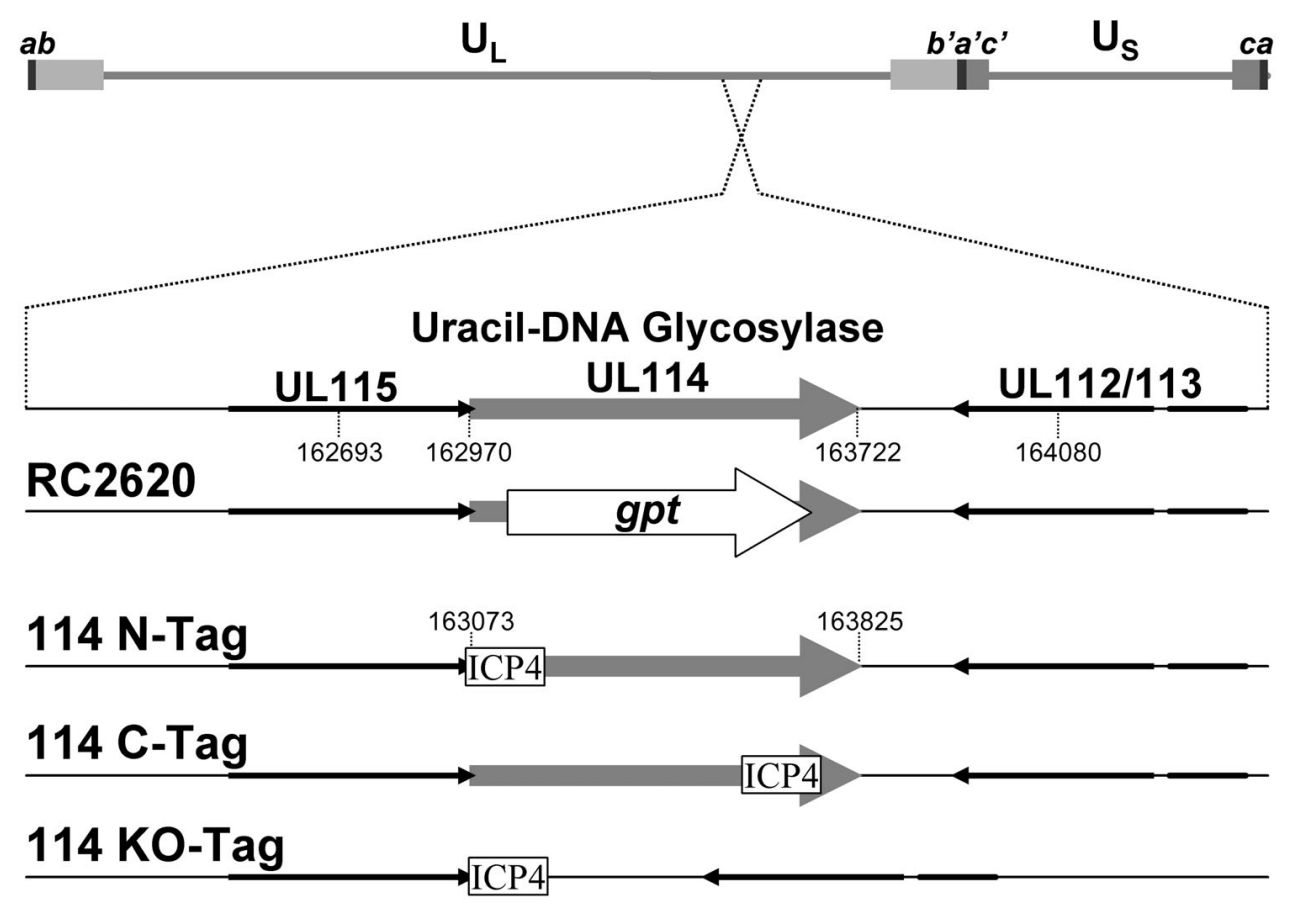
**Recombinant viruses. **The top line represents the CMV genome with the region surrounding UL114 expanded below. The second line represents the structure of the region in the parent virus (AD169). The third line labeled "RC2620" depicts the 1.2 kb insertion containing the *E. coli gpt *gene (white arrow) that replaces most of the UL114 ORF. The final three lines represent the same region in Towne and depict the placement of the 35 aa ICP4 epitope tags in the ORF. The entire ORF was also deleted in Towne as a control and resulted in the same slow replication phenotype as was observed in the AD169 strain.

**Figure 2:**
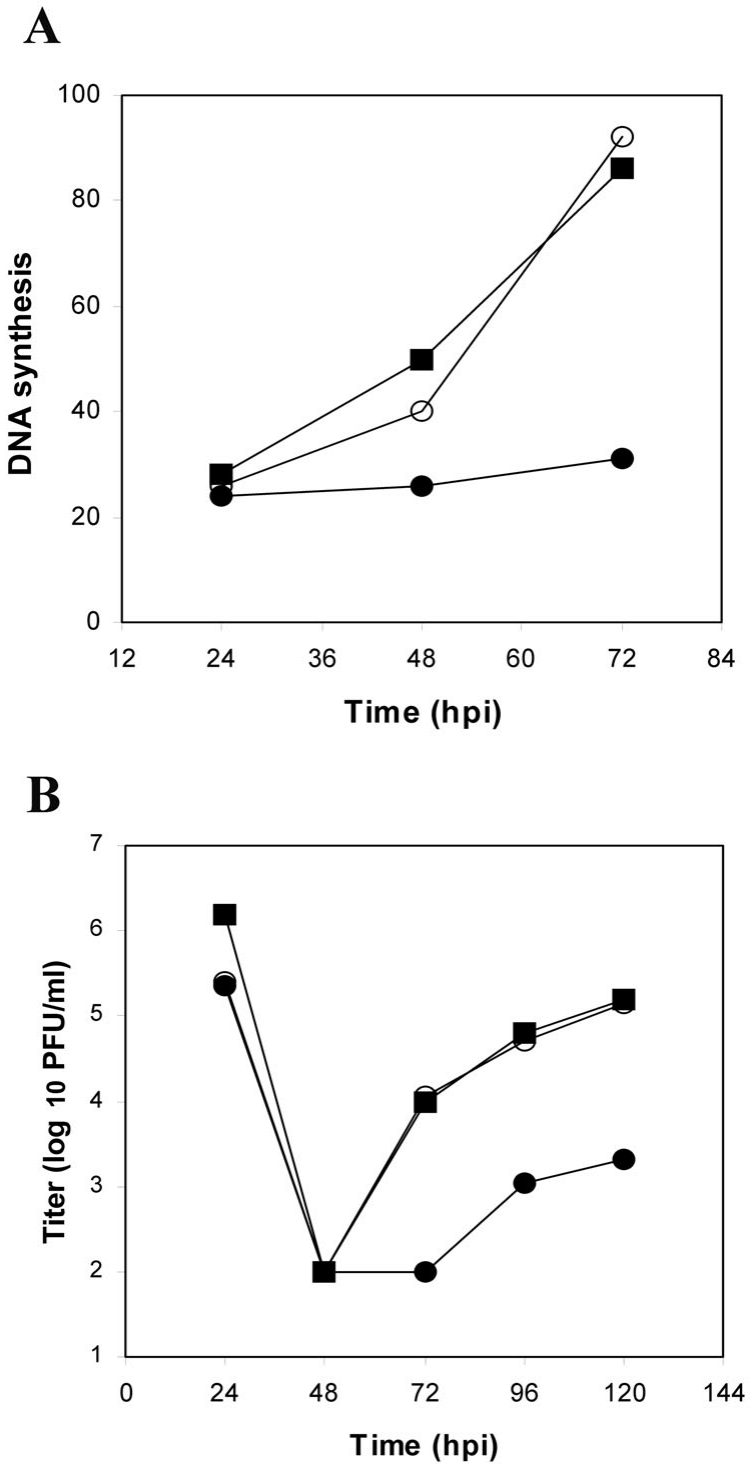
**Repair of RC2620. **(A) HEL cells were infected at an MOI of 5 PFU/cell and total DNA was harvested at the indicated times. The quantity of viral DNA for AD169 (black squares), RC2620 (black circles), and RQ2620 (open circles) were determined by dot blot hybridization as described in materials and methods. (B) Titers of AD169 (black squares), RC2620 (black circles), and RQ2620 (open circles) are shown. The time point at 0 hpi represents the titer of the input virus.

Recombinant viruses with deletions in *UL114 *express early gene products with normal kinetics, yet exhibit a marked delay in the onset of DNA synthesis [6,31]. This phenotype was assigned to *UL114*, since two independent isolates of the recombinant virus exhibited the same phenotype. To formally ascribe the observed phenotype to this locus, the lesion was repaired with an Eag I DNA fragment (AD169 coordinates 162693–164080) that spans the deletion in the mutant virus (Fig. 1). Plaques resistant to high concentrations of xanthine were isolated and were shown to have restored the deleted sequences as determined by Southern analysis (data not shown). Kinetics of viral DNA synthesis were examined in HEL cells infected with the parent virus, the mutant (RC2620) and the rescued virus (RQ2620) to determine if the restoration of the *UL114 *locus reverted the phenotype of delayed DNA synthesis. As observed previously, the mutant exhibited very little DNA synthesis in the first three days of infection (Fig 2A). In contrast, the rescued virus appeared to synthesize DNA with the same kinetics as the parent virus suggesting that the defect was due to the engineered mutation rather than to mutations elsewhere in the genome. These data were confirmed in HEL cells in an experiment in which single-step replication kinetics were examined. Delayed viral replication was observed in the mutant virus, whereas, no difference was observed between the *wt *virus and the recombinant virus in which the *UL114 *lesion was repaired (Fig. 2B). Thus, two facets of the described phenotype (DNA synthesis and replication kinetics) were reverted upon restoration of this gene and we formally assigned this phenotype to the engineered mutation. This phenotype was also reproduced in Towne strain of CMV when the UL114 open reading frame was disrupted.

### Complementation of the UNG deficient mutant in *trans *and the effect of uracil content on the phenotype

Previous work demonstrated that virion DNA from the mutant virus contained modestly elevated levels of uracil compared to the *wt *virus, which is a predicted phenotype [31]. Thus, it is possible that the delay in DNA synthesis simply reflects the time required to repair misincorporated uracil residues in the input viral genomes, and once this is accomplished, DNA synthesis proceeds normally. To test this hypothesis, a cell line that could complement the mutant virus in *trans *was constructed by methods described previously [32]. Virus stocks produced in the complementing cell line (HL114) were determined to possess normal levels of uracil, suggesting that the cell line was able to compensate for the deficiencies in the deletion mutant (data not shown). Thus, subsequent infection of HEL cells with these complemented virus stocks should reveal effects that are related to the genetic differences of the viruses, rather than the physical characteristics of the input genomes.

**Figure 3:**
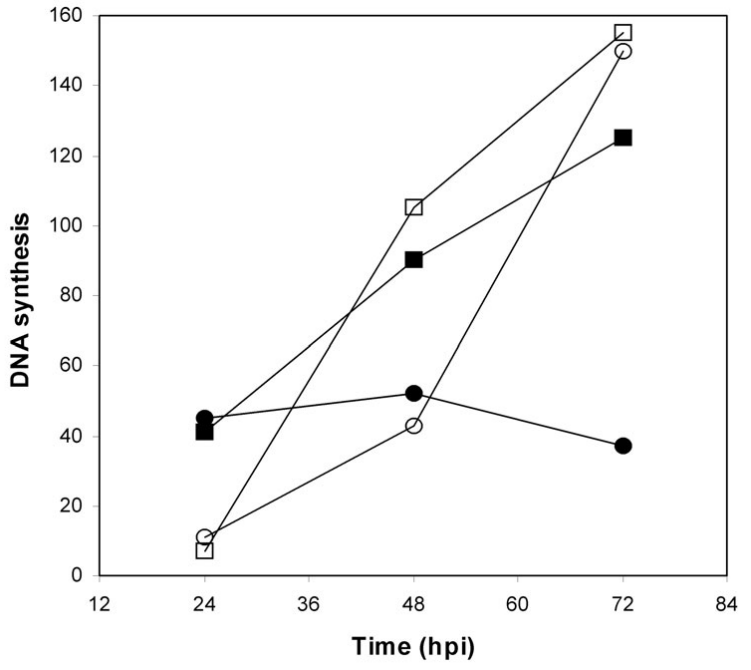
**Kinetics of DNA synthesis and viral replication in complementing cells. **Virus stocks of the parent virus and the mutant virus were produced in the complementing cell line (HL114) and used to infect either HEL cells or IHL114 cells at an MOI of 5 PFU/cell. Circular and square symbols represent quantities of DNA from RC2620 and the repaired virus respectively while solid and open symbols represent DNA isolated from HEL cells and HL114 cells respectively. The average of triplicate values are shown.

Complemented virus stocks were used to infect both HEL cells and HL114 cells at an MOI of 5 PFU/cell and kinetics of viral DNA synthesis were determined. In HEL cells, the mutant virus failed to induce detectable DNA synthesis at 72 hpi, whereas cells infected with repaired virus synthesized large quantities of viral DNA (Fig. 3). A similar result was obtained when uncomplemented virus stocks were used to infect these cells (data not shown). This suggested that the defect in DNA synthesis was likely related to a deficiency in pUL114 rather than the uracil content of the input viral genomes. As a control, both viruses were used to infect the complementing cells and both viruses produced similar quantities of DNA by 72, hpi, indicating that pUL114 supplied in *trans *could complement the observed defect in DNA synthesis. The complementation did not appear to be complete however, and there does appear to be a slight lag in DNA synthesis by the mutant virus. These results were confirmed by titering progeny virus at 96 hpi, when the mutant virus exhibits titers that are more than ten-fold lower than the parent virus in primary fibroblasts. Infection of complementing cells produced indistinguishable titers of both the mutant and restored viruses, while titers of the deletion virus were reduced more than ten-fold in primary fibroblasts (data not shown). Thus, the physical characteristic of the deletion mutant's genome appear to be unrelated to the observed phenotype and it appears more likely that the observed defects are due to a deficiency in pUL114 during the lytic replication cycle.

### Construction of epitope tagged viruses

**Figure 4:**
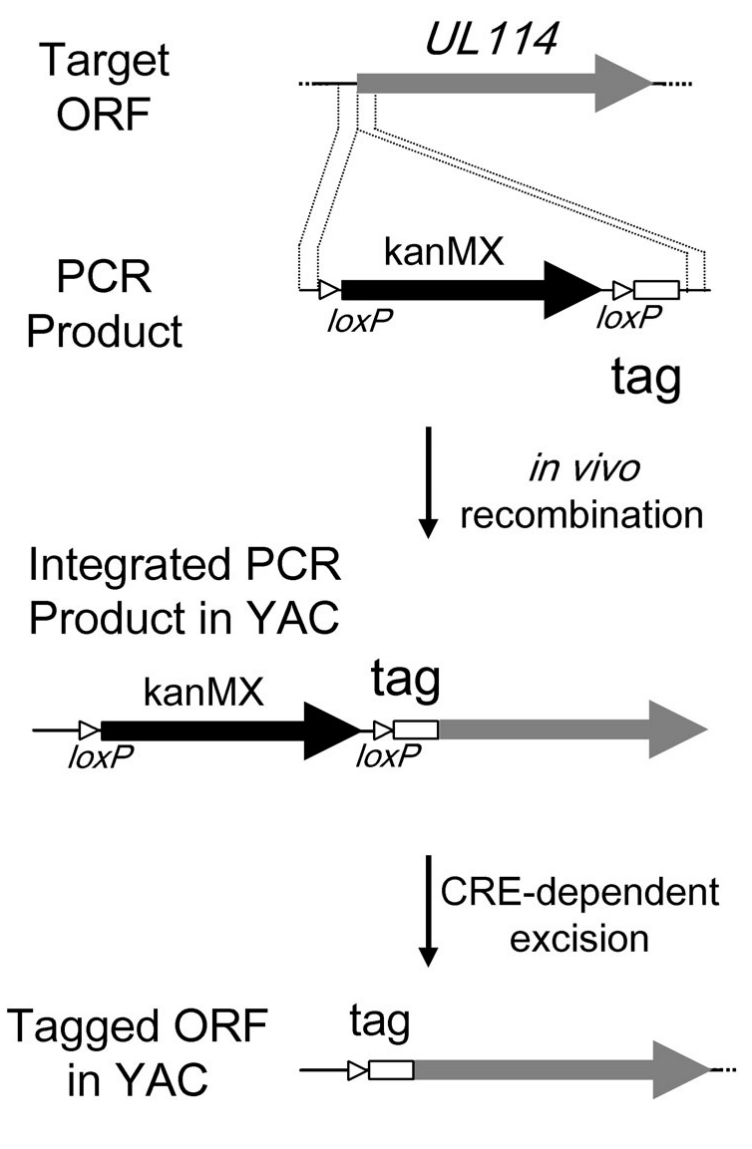
**Rapid epitope tagging strategy in yeast. **The top line represents the target ORF in the context of a large yeast plasmid or YAC. Line 2 shows a PCR product containing the epitope tagging cassette with 40 bp targeting sequences homologous to the regions designated by the dashed lines. Line 3 shows the site-specific integration of the cassette resulting from homologous recombination in yeast. The final line represents UL114 in the YAC with an in frame 35 aa amino terminal insertion containing the ICP4 epitope and a single *loxP *site. This strategy can be used to place the epitope tag anywhere in the ORFs on the YAC by changing the targeting sequences on the PCR primers.

To investigate a potential role for pUL114 in viral DNA replication, it was necessary to characterize the expression and intracellular localization of this gene product during the replication cycle. Site directed mutagenesis in very large constructs is difficult to accomplish using standard techniques, so a rapid method for epitope tagging viral genes was developed. Homologous recombination in *Saccharomyces cerevisae *was conducted by methods similar to those described earlier in yeast artificial chromosomes [19]. A previous report described a method for recycling the KanMX selectable marker in yeast, through the induction of CRE recombinase that resulted in the *loxP *dependent excision of this marker. This construct was modified such that a precise deletion of the marker would yield an in frame 35 aa insertion including the ICP4 epitope tag. Amplification of pkanMX-ICP4 allowed the insertion of this epitope tag anywhere in the viral cosmid with primers containing 40 bp 5' extensions to target the desired locus in the DNA (Fig. 4). This technique was used to construct three cosmids in which *UL114 *was tagged at the amino (UL114NTAG) and the carboxyl (UL114CTAG) termini, as well as the precise replacement of UL114 with the 35 aa ORF containing the epitope tag (UL114KO) (Fig. 1). Resulting cosmids were used in a standard cotransfection to generate three tagged recombinant viruses by methods described previously [15].

### Localization to replication compartments and association with ppUL44

**Figure 5:**
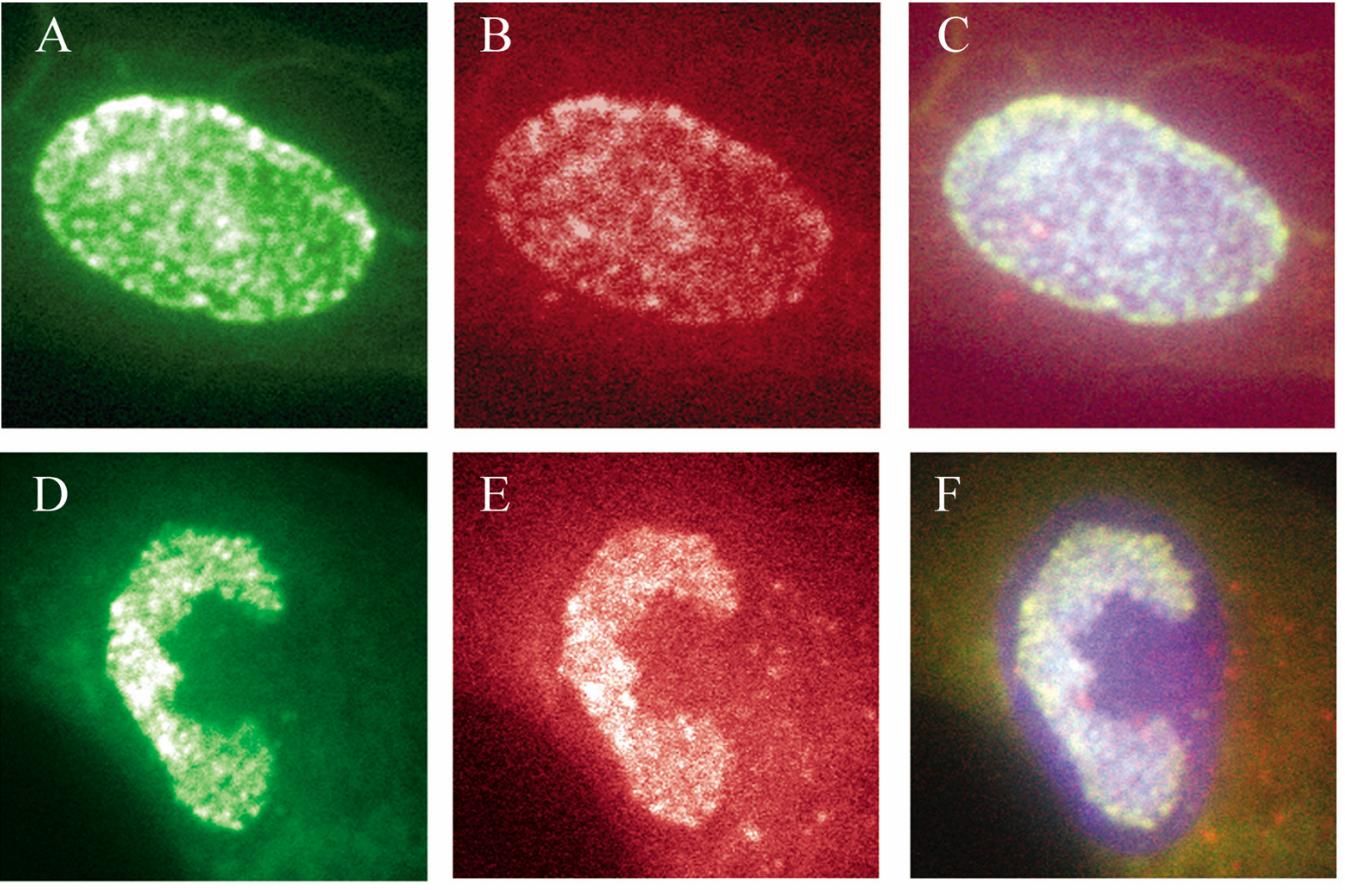
**Localization of pUL114 in infected HEL cells. **Cells were infected with a recombinant virus with an epitope tag in the carboxyl terminus of UL114. Monolayers were fixed and stained with an anti-ppUL44 monoclonal antibody (FITC) and an anti-ICP4 mouse monoclonal antibody (Texas Red). Cells were fixed at 48 hpi and images of FITC, Texas Red, and a merged image with DAPI are shown(A-C). Cells were fixed at 72 hpi and images of FITC, Texas Red, and a merged are shown (D-F). All images were captured digitally and prepared in Adobe Photoshop.

Previous work used immunofluorescence microscopy to examine the nature and distribution of CMV replication components at various times in the virus life cycle [29]. This work suggested that various members of the viral replication complex, including ppUL44, the DNA polymerase processivity factor, localize into specific replication compartments in patterns that are characteristic of a given point in the replication cycle. In light of the putative role of the *UL114 *gene product in viral DNA replication, similar studies were undertaken, using the epitope-tagged viruses described above to determine the location of pUL114 in infected fibroblasts. HEL cells were infected with the recombinant viruses and were examined by fluorescence microscopy using anti-ICP4 and anti-UL44 monoclonal antibodies. At 48 hpi, ppUL44 localized to the nucleus in small foci in a pattern that was very similar to that for pUL114 (Fig 5A–C). By 72 hpi, epitope tagged pUL114 expressed from the CTAG virus partitioned to the replication compartments within the nucleus as defined by ppUL44 staining (Fig. 5D–F) and light punctate cytoplasmic staining was also observed in some cells. The recombinant UL114 NTAG virus did not exhibit the strong nuclear localization observed with UL114 CTAG and it is possible that fusing the ICP4 epitope to this part of the molecule may have interfered with its normal localization (data not shown).

**Figure 6:**
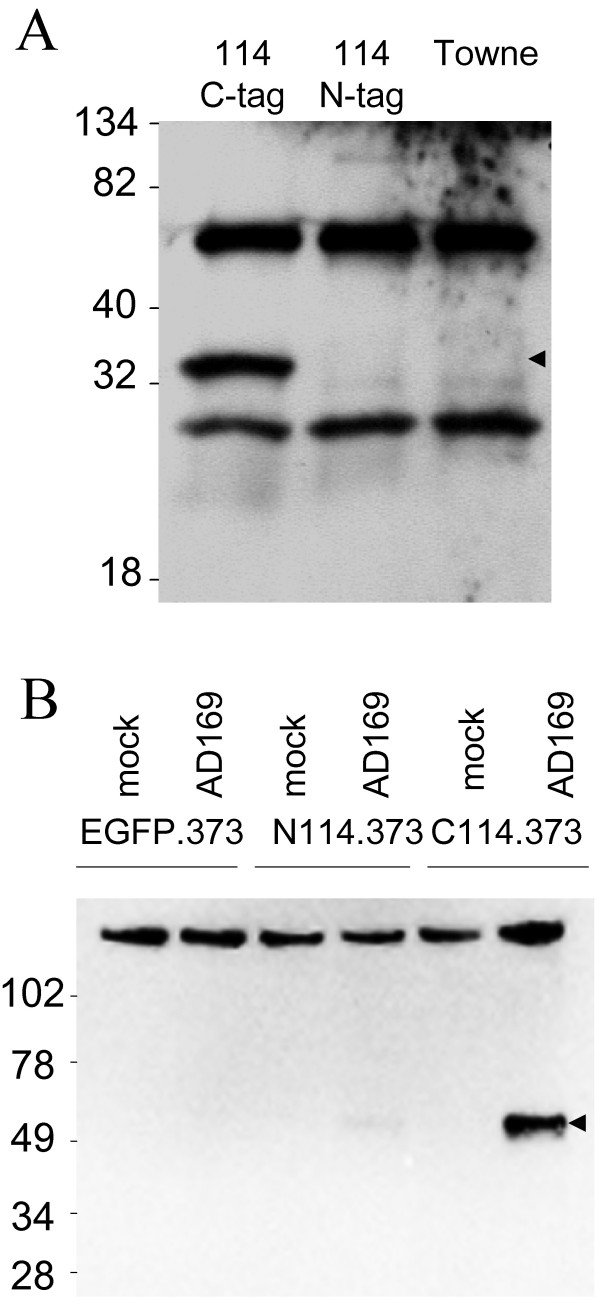
**Coprecipitation of pUL114 and ppUL44**. (A) Primary foreskin fibroblast cells were infected either with ICP4-tagged recombinant viruses or Towne at an MOI of approximately 1 PFU/cell. Cells were lysed at 48 hpi, and extracts were immunoprecipitated with a monoclonal antibody to ppUL44 and separated on an SDS-PAGE gel. Proteins were transferred to a membrane and a monoclonal antibody to the ICP4 epitope was used to detect coprecipitated poteins in the immunoblot. (B) EGFP.373 and C1-114.373 cells were infected with AD169 at an MOI of 2 PFU/cell and harvested at 24 hpi. Fusion proteins were precipitated with a monoclonal antibody to EGFP, separated on non-denaturing SDS PAGE gels, transferred to nitrocellulose, and immmunoblotting was performed with monoclonal antibody to ppUL44. Arrows designate the specific bands.

The localization pattern exhibited by the tagged versions of pUL114 suggested that it might be physically interacting with the viral DNA replication machinery. We hypothesized that pUL114 might interact with ppUL44 analogous to the UNG2 interaction with PCNA that occurs the human DNA replication complex [28]. Extracts of cells infected with the epitope tagged viruses and a *wt *virus were immunoprecipitated with a monoclonal antibody to ppUL44. Precipitated proteins were separated on denaturing polyacrylamide gels, transferred to nitrocellulose and a monoclonal antibody specific for the ICP4 epitope was used to detect the tagged pUL114 molecules. A protein with a predicted molecular weight of 32 kDa was specifically detected from the recombinant virus in which pUL114 was tagged at the carboxyl terminus (Fig. 6A). A very light band with the same migration rate was detected from UL114 NTAG-infected cells upon long exposure, consistent with its reduced localization to the nucleus. No specific species were detected in extracts prepared from the *wt *virus. The reverse experiment was performed with pUL114-EGFP fusion proteins that were precipitated with a monoclonal antibody specific for GFP and the monoclonal antibody to ppUL44 was used to detect the coprecipitated protein. This experiment confirmed the earlier result and demonstrated that it was also possible to specifically coprecipitate ppUL44 with pUL114 fusion proteins (Fig. 6B). Consistent with the previous result, the coprecipitation appeared to be less efficient for pUL114 labeled at the amino terminus.

**Figure 7:**
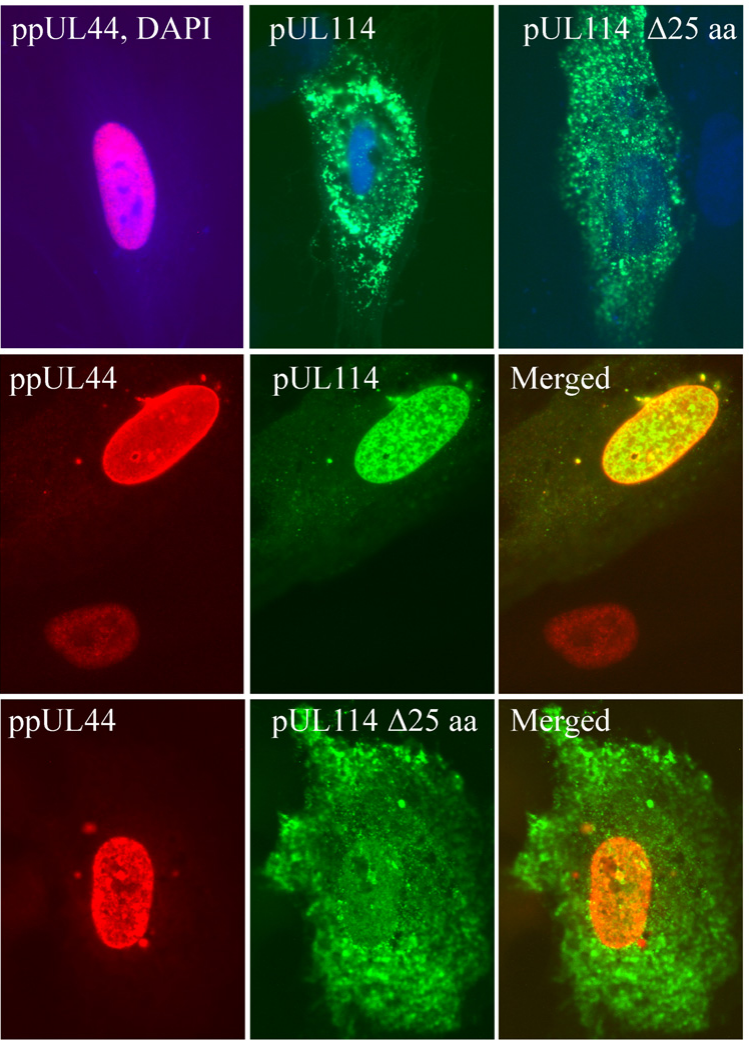
**Recruitment of pUL114 to the nucleus by ppUL44**. Plasmids expressing ppUL44 or pUL114-EGFP fusion proteins were transfected into primary fibroblast cells and visualized by immunofluorescent staining. In the first row of images, ppUL44 stained with Texas Red exhibited strong nuclear localization as evidenced by the colocalization with DAPI in the merged image (violet). The pUL114-EGFP fusion protein and a similar protein containing a 25 aa amino terminal deletion (green) both localized to the cytoplasm and are shown merged with DAPI staining (blue). In the second row of images, the coexpression of ppUL44 (Texas red), pUL114-EGFP (green) and a merged image show that ppUL44 can recruit pUL114 to the nucleus. In third row of images ppUL44 (Texas red) and pUL114-EGFP containing a 25 aa amino terminal deletion (green) did not colocalize to the nucleus when co-expressed.

To confirm these results, plasmids expressing ppUL44 (pMP62) and pUL114 with a carboxyl terminal EGFP tag were transfected into monolayers of primary foreskin fibroblast cells. In cells transfected with pMP62 alone, ppUL44 localized exclusively to the nucleus and is shown merged with DAPI image (Fig 7A), which was similar to the localization observed in infected cells early in infection. Cells expressing either the full length pUL114-EGFP fusion protein (pMP39), or the fusion protein in which aa 3–24 were deleted from pUL114 (pMP41) exhibited punctate cytoplasmic fluorescence (Fig 7B, C). This localization pattern was distinct from the nuclear staining observed with the UL114 CTAG recombinant virus. However, when ppUL44 and full length pUL114 fusion proteins were coexpressed in the same cell, pUL114 was recruited to the nucleus with ppUL44 (Fig 7D–F), consistent with its nuclear localization in the context of infected cells. A small quantity of ppUL44 also appeared to localize to a subset of the cytoplasmic punctae containing pUL114. Deletion of aa 3–24 from the pUL114 fusion protein eliminated its recruitment to the nucleus by ppUL44, suggesting that this domain is required for the interaction ppUL44 (Fig 7G–I). This interpretation of the data is consistent with the impaired nuclear localization observed with UL114 NTAG-infected cells, in which the amino terminal domain of pUL114 was altered through the addition of the ICP4 epitope tag (data not shown). Also consistent with this result, is the inefficient coprecipitation of ppUL44 with pUL114 fusion proteins when the tags were fused to the amino terminus (Fig. 6). These data suggest that these proteins associate in a manner that is dependent on aa 3–24 of pUL114, and independent of other viral proteins or viral DNA. These experiments do not, however, eliminate the possibility that they might associate in an indirect manner through cellular proteins.

### Characterizing the defect in DNA synthesis

**Figure 8:**
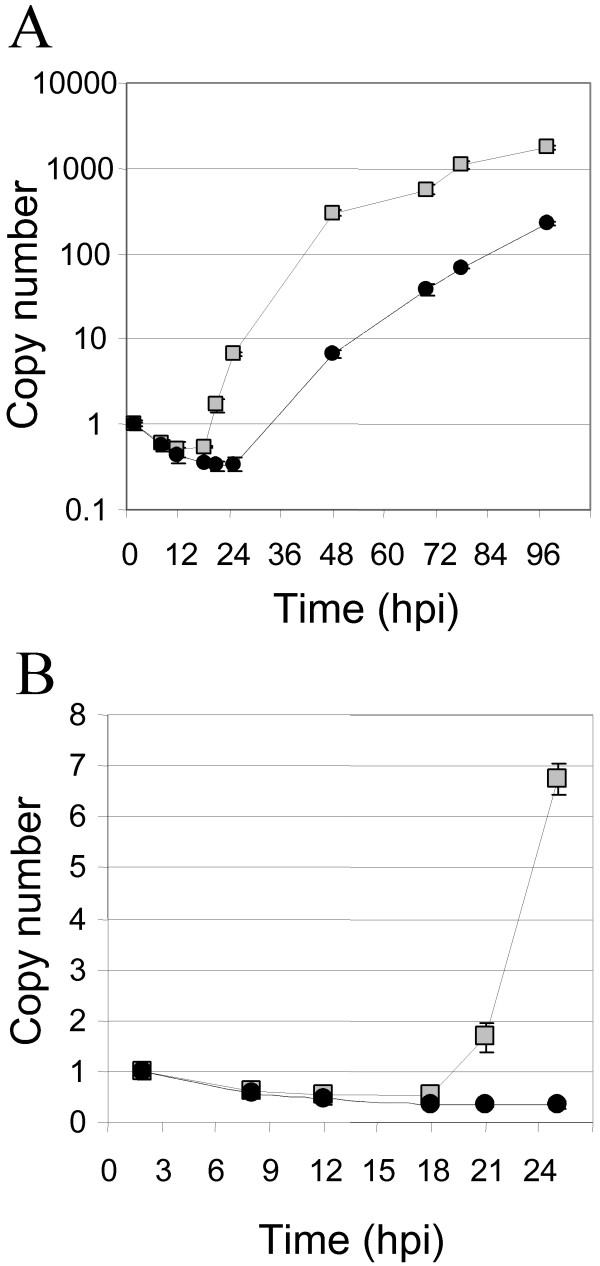
**Defects in DNA synthesis associated with pUL114. **Triplicate wells of HEL cells were infected at an MOI of 0.01 with Towne (black circles) or the isogenic deletion virus, UL114 KO tag, (shaded squares). Total DNA was harvested at the indicated times, and the genome copy number was determined with a TaqMan assay using a standard curve of virion DNA. Copy number was normalized to the quantity of input genomes determined at 2 hpi with error bars representing the standard deviation of the triplicate samples. (A) The log of the accumulated viral DNA copy number is shown versus time post infection. The *wt *exponential rate of accumulation (r) was determined to be approximately 0.2 h^-1^, whereas the copy number of the mutant expanded only at a rate of about 0.1 h^-1^. (B) Data for the first 24 h replotted on a linear scale show the delayed onset of DNA synthesis during the first duplication of the viral genome.

The localization of pUL114 to replication compartments, and its apparent association with ppUL44, which is known to interact with the DNA polymerase [9] imply that this molecule is part of the viral DNA replication complex. This interpretation of the data is consistent with the observed phenotype of delayed DNA synthesis in the UL114 deletion virus [6,31], and is also consistent with results reported for the human UNG2 that has been shown to localize to replication complexes [28]. If this assumption is correct and the viral UNG is an important part of the replication complex, then the defect in viral DNA synthesis should be apparent throughout the viral DNA replication process. To characterize the affect of pUL114 on DNA synthesis, triplicate monolayers of replicating primary foreskin fibroblasts were infected with either Towne, or an isogenic recombinant virus without *UL114 *and the accumulation of viral DNA was quantified with a TaqMan-based assay. Input copy number following infection was determined at 2 hpi and yielded average values of 4.2 × 10^4 ^and 2.3 × 10^4^, for the wt and mutant viruses respectively with standard deviations of <15% for both values. During the course of infection, genome copy number was determined in total DNA and the data were normalized relative to the input copy number (Fig. 8). During the first 18 h of infection, copy number of the *wt *and deletion virus genomes decreased at the same rate with a half-life of approximately 8 h (Fig 8B). This is consistent with data presented earlier, which suggested that increased uracil levels did not substantially affect genomic integrity and were unlikely to be responsible for the observed defects in DNA synthesis. This analysis also revealed two features of the defect in DNA synthesis. First, the accumulation rate of viral DNA synthesis was significantly reduced in the recombinant virus with a deletion in *UL114 *(Fig. 8A). A 7-fold increase in copy number was attained in the parent virus at 25 hpi, but this same level was not achieved in the mutant until 48 hpi. By this time, the *wt *virus had attained a 300-fold amplification of the input genome, which was not attained by the mutant even after an additional 48 h of incubation. Exponential growth rates were calculated from curves fitted to the experimental data for both viruses. The *wt *rate (r) was determined to be approximately 0.2 h^-1^, whereas the copy number of the mutant expanded at a rate of about 0.1 h^-1^. This decreased rate of DNA accumulation is consistent with the observed decrease in viral DNA described previously [31] and also with the data showing a defect in the transition to late phase DNA synthesis reported recently by Courcelle et al. [6]. A second defect in DNA synthesis was also observed. The initial doubling of the *wt *genome was detected at 21 hpi and the copy number increased exponentially to a 7-fold increase by 25 hpi (Fig. 8B). During this period of time, no increase in the copy number of mutant virus genomes was observed. Thus, the initial phase of DNA synthesis also appears to be compromised in the absence of pUL114, despite the fact that early genes are expressed at normal levels at this point in time [29,31]. If viral DNA synthesis in the mutant had initiated at the same time as the parent virus, the increased copy number should have been easily detectable by 25 hpi, even at the reduced rate of accumulation we report here. Thus, either the initiation or the early theta-type DNA replication postulated for this family of viruses appears to be compromised in absence of pUL114. These data suggest that pUL114 acts during both the onset and the subsequent expansion phase of viral DNA synthesis and suggests that this gene product functions as part of the viral DNA replication machinery.

## Discussion

Perhaps the simplest explanation of the observed phenotype associated with *UL114 *deletion viruses is that the recombinant virus fails to remove uracil residues from its genome and that these lesions decrease genome stability and impede DNA synthesis. Two lines of evidence argue against this interpretation of the data. First, input genomes of the recombinant virus in infected cells appeared to be as stable as the *wt *genomes in infected cells and had similar initial half lives (Fig 8B). Second, the complementing cell line reduced the uracil content of the mutant genomes to levels indistinguishable from the parent virus, yet the observed phenotype of these complemented virus stocks in non-complementing cells was unaffected. Thus, it appears that the viral UNG plays a more direct role in the synthesis of viral DNA. However, these data do not exclude the possibility that the removal of uracil may be important late in infection. We suggest that the HCMV UNG2 homolog functions as part of the DNA replication machinery and that it significantly accelerates the synthesis of genomic DNA.

The parallels between this system and the recent results for human UNG2 are striking. PCNA and ppUL44 are thought to perform a similar function and associate with human DNA polymerase δ and the HCMV DNA polymerase, respectively. Despite the fact that these processivity factors do not share significant aa sequence homology and exhibit different 3-D structures [1], they retain interactions with their respective DNA polymerases [21], as well as an association with their respective UNG homologs. The fact that the amino terminal domains of both pUL114 and UNG2 are required to mediate these interactions suggests that this might be a common feature among all UNG2 homologs. This relationship is also conserved in vaccinia virus where the viral UNG2 homolog (D4R) was shown to physically associate with the A20R DNA polymerase processivity factor [14]. In this system, the viral UNG was shown to be essential for viral DNA synthesis, and this requirement was unrelated to the ability of the molecule to excise uracil [7]. A potential role for UNG in DNA replication was also noted in Epstein Barr Virus where the UNG2 homolog (BKRF3) increased the efficiency of replication of a transfected plasmid containing the origin of replication [10] and was absolutely required when the core essential genes were supplied on a set of cosmid clones [11]. Less analogous but equally compelling, is the recruitment of UNG2 to the preintegration complex in HIV and its specific interaction with both the integrase as well as the reverse transcriptase [33]. The conserved relationship between UNG2 homologs and DNA replication complexes in these diverse systems suggests that it performs a conserved function in mammals. It is unclear if this function is related to UNG enzymatic activity, and it is likely that these molecules perform an additional function replication that remains uncharacterized. This view is supported by the fact that the UNG enzymatic activity can be eliminated without severely affecting the replication of vaccinia virus, whereas larger mutations are lethal [7]. A specialized role for UNG2 has also been proposed in mammalian systems since UNG^-^/UNG^- ^mice are viable and do not exhibit the phenotype of highly elevated mutation frequency that would be predicted by earlier studies in prokaryotes and *Sacharomyces*. Information garnered in future studies with HCMV will be particularly helpful in shaping our understanding of the function of UNG2 in the DNA replication foci of mammalian cells. The unique phenotype associated with pUL114 in HCMV infection and the fact that this simple system closely resembles that in humans make it an attractive system to probe the unique function of mammalian UNG2 homologs in DNA synthesis.

In HSV, the deletion of the UNG homolog (UL2) affects the ability of the virus to replicate in mice, particularly the CNS. The deletion of UL2 resulted in a 100,000-fold reduction in the neuroinvasiveness and may represent a potential attenuating mutation in candidate vaccines [34] Previous studies with UNG deletion mutants in HSV were not shown to affect replication in tissue culture, they replicated to lower titers *in viv*o and were orders of magnitude less neuroinvasive than control viruses [34]. To investigate the possibility that the phenotype might be more pronounced *in vivo*, we infected human fetal retinal tissue implanted in a SCID-hu mouse [2,16]. In this model, a deficiency in pUL114 resulted in a decreased infection rate (P = 0.015) as well as significantly reduced titers in infected animals (P = 0.0063). However, the observed defects in vivo were not more pronounced that the replication defects in cell culture and were not similar to results observed with HSV.

## Conclusion

The work presented here suggests that pUL114 is part of the DNA replication machinery and that it significantly accelerates the synthesis of genomic DNA. This interpretation of the data is consistent with the early expression kinetics and the nuclear localization exhibited by this molecule in infected cells, which are both predicted characteristics of an enzyme presumed to act in DNA repair. Equally consistent is the observed intranuclear localization to viral replication compartments at a time when viral DNA synthesis is known to occur [29]. The fact that pUL114 appears to associate with ppUL44 is intriguing, because of the central role that ppUL44 plays in the synthesis of viral DNA [9,20,21]. These data taken together with the observed defects in the onset and expansion of viral DNA synthesis suggest that it functions as part of the DNA replication machinery.

We propose a model in which pUL114 functions as part of the viral DNA polymerase complex and is required for the efficient establishment and expansion of viral DNA synthesis. Results presented here suggest that the performance of the DNA replication machinery is significantly impaired without pUL114. The precise mechanism that this molecule uses to affect DNA synthesis is unclear but it may or may not be related to its ability to excise uracil from DNA. The interaction with ppUL44 suggest that this molecule might be close to the replication forks where it might help destabilize double stranded DNA through a scanning and pinching base flipping mechanism similar to that described for the human homolog [12]. Additional experiments in this system will be required to determine the correlation between uracil excision activity and the efficiency of viral DNA replication.

The evolving view of UNG function in the life cycle of viruses increases its appeal as a target for antiviral chemotherapy, particularly in poxviruses where it is essential for virus replication. This approach may also be valuable in herpesviruses given its proximity to the replication complex as well as its important role *in vivo*. It is certainly possible to obtain specific inhibitors of viral UNG molecules based on their ability to block the enzyme's ability to excise uracil, however at present, it is unclear that this enzymatic activity is responsible for the interesting affects observed both *in vitro *and *in vivo*. Rational drug strategies should be possible, but their development is dependent upon a better understanding of the biological functions of this molecule in virus replication.

## Methods

### Plasmids

Construction of pON2619 and pON2620 were described previously [31]. To construct a retroviral vector, a 1782 bp EcoRI fragment (coordinates 163071 to 164853 AD169 genome) containing the UNG open reading frame was inserted into the MfeI site in pLXIN (Clontech, Palo Alto, CA) to yield pON2159. EGFP fusion constructs were constructed by amplifying an 800 bp DNA fragment containing the UL114 open reading frame using the forward primer 5'-GGA CTC AGA TCT ATG GCC CTC AAG CAG TGG ATG-3' and the reverse primer 5'-GTC GAC TGC AGA GAA TCT CCC ACA GAG TCG CCA GTC C-3'. The resulting fragment was purified from an agarose gel and cloned into the Bgl II and Pst I sites of pEGFPC1 and pEGFPN3 (Clontech, Palo Alto, CA) to generate plasmids pEGFPC1/UL114 and pEGFPN3/UL114. Plasmids pMP39 and pMP41 were constructed by amplifying with forward primers 5'-ATG GCC CTC AAG CAG TGG-3' and 5'-ATG GCC GCT CGC GTG TTT TGT CTG AGC-3' respectively, with reverse primer 5'-TCA TCT GAG TCC GGA CTT GTA CA-3' using pEGFPN3/UL114 as a template and cloning into pcDNA3.1. The resulting plasmids were sequenced and express proteins of the predicted molecular weight. The UL114 open reading frame in pMP41 contains a deletion of aa 3 to 24. Primers 5'-CAC CAT GGA TCG CAA GAC GCG C-3' and 5'-CTA GCC GCA CTT TTG CTT CT-3' were used to amplify UL44 and the PCR product was cloned into a eukaryotic expression vector to yield pMP62. The plasmid pUG6 contains a recyclable genetic marker for site directed mutagenesis in yeast [13]. This cassette was amplified with the forward primer 5'-CAG GTC GAC AAC CCT TAA TAT AAC TTC GTA TAA TGT ATG CTA TAC GAA GTT ATT AGG TCT AGA GAT CTG TTT AGC TTG C-3' and the reverse primer 5'-TCC TGG AGC TCG ATC TCC TGC TGC ATC TGC TGC ATC ATC ATA TTC ATC ACC TAA TAA CTT CGT ATA GCA TAC ATT ATA CGA AGT TAT ATT AAGGGT TCT CG-3'. The resulting product was TOPO-cloned into pcDNA3.1 (Invitrogen, Carlsbad, CA) to yield pKan-ICP4 and used as a template for subsequent amplifications. The *Sma *I – *Sca *I fragment of pRS413, which contains ARS4, CEN6 and the HIS3 selectable marker, was ligated into the *Xmn*I site of pACYC184. A single *Eco*RI site in the resulting intermediate construct was converted to a unique *Pac*I site by ligation to *Eco*RI *Pac*I adapters to produce pACYC ars cen. PacI fragments from cosmids described previously [15] were cloned in the PacI site for subsequent experiments.

### Mutagenesis in Yeast

PCR products for site directed mutagenesis were generated using the forward primer 5'-AGG TCG ACA ACC CTT AAT ATA ACT-3' and reverse primer 5'-TCC TGG AGC TCG ATC TCC TGC TGC AT-3' and were targeted by adding 40 bp of homologous sequence to the 5' end of each primer. PCR products were cotransformed by a standard lithium acetate protocol with target viral cosmids in *Saccharomyces cerevisiae *strain CGY2570 carrying plasmid pSH47 [13] which expresses CRE recombinase under control of the GAL promoter. Recombinants were selected on yeast complete medium plates containing 400 μg/ml G418 and the selectable marker was excised through the galactose-dependent expression CRE recombinase to yield 35 aa in frame insertions containing the HSV ICP4 epitope tag.

### Cells and virus

Primary human foreskin fibroblast (HFF) cells and human embryonic lung (HEL) cells were grown in monolayer cultures in Dulbecco's modified Eagle medium (Gibco BRL, Gaithersberg, MD) supplemented with 100 units/ml penicillin G, 100 μg/ml streptomycin sulfate and 10% fetal bovine serum (FBS). Parental virus (AD169) was obtained from the ATCC and virus stocks were obtained and titered as described previously [40]. Cell lines expressing EGFP fusion proteins were constructed by transfecting 10 μg of linearized pEGFPC1/UL114, pEGFPN3/UL114 or pN3EGFP inU373 cells with Lipofectin (Gibco BRL, Gaithersberg, MD) according to the manufacturers recommendations. Stably transfected C1-114.373, N3-114.373 and EGFP.373 cells were selected with 1 mg/ml G418, and resulting colonies were isolated and frozen at passage 5. The construction and propagation of RC2620 as well as the production of high MOI growth curves were described previously [31]. The construction of RQ2620 was performed as described previously [31] and the resulting repaired virus was plaque purified 3 times after it was shown to be free of the contaminating parent virus by Southern analysis. Epitope tagged viruses were constructed by cotransfecting a set of 8 cosmids derived from the Towne strain of HCMV [15], including one cosmid that was subjected to site directed mutagenesis in yeast as described above. The epitope tagged viruses replicated to high titers in HFF cells and do not appear to be replication impaired.

### Construction of HL114 cells

pON2159 was tranfected into PA317 cells to produce defective retrovirus stocks that were subsequently used to transduce the UL114 gene into low passage primary HEL by methods described previously [32]. Transduced cells were selected with 400 μg/ml G418 starting at 24 hpi. Surviving cells were passaged in G418 and were used as a mixed population.

### DNA synthesis kinetics

Confluent monolayers of HFF cells in 6-well cluster dishes were infected at an MOI of 5 PFU/cell with either the AD169, RC2620, or RQ2620. Total DNA was extracted as described previously [31], diluted and transferred to a Hybond N+ membrane in a dot-blot manifold and probed with a plasmid containing viral sequences (89797-94860 in the AD169 genome). The resulting film was captured digitally and quantified with Scan Analysis (Biosoft, Cambridge, UK).

### Determination of genome copy number

Towne and an isogenic recombinant virus containing a deletion in UL114 were used to infect dividing HFF cells at an MOI of 0.02 PFU/cell in 12-well plates. Monolayers were rinsed three times at 2 hpi and supplemented with fresh media. At harvest, monolayers were rinsed twice with fresh media, and frozen at -80°C in a final volume of 0.2 ml. Total DNA was extracted using a QIAamp DNA blood minikit according to the manufacturers recommendations (Qiagen, Valencia, CA). Copy number was determined using the ABI PRISM 7700 sequence detection system and TaqMan Universal PCR Master Mix. Forward primer (50 nM), 5'-CCG AGG TGG GTT ACT ACA ACG-3', reverse primer (300 nM), 5'-GGA AGG GTA GAG GCT GGC A-3', and fluorogenic probe (75 nM), 5' FAM-CCC CGT GGC CGT GTT CGA CT-3' TAMRA were used in a 50 μl reaction volume with conditions as follows: 2 min at 50 ?C, 10 min at 95 ?C, and 40 cycles of (15 sec at 95 ?C, 1 min at 60 ?C,). The DNA templates from triplicate wells were analyzed in a volume of 5 μl per reaction. Copy number was compared to a standard curve generated from HCMV genomic DNA.

### Immunofluorescence microscopy

Immunofluorescence staining was performed as previously described [37]. 8-well chamber slides of confluent HFF cells (LF1043) were infected with recombinant HCMV strains UL114 NTAG, UL114 CTAG, and Towne (control) at an MOI of 0.5 PFU/cell. Slides were washed once with PBS, fixed with 1% formalin for 15 min, and washed again three times with PBS + 0.2% BSA. Permeablization was performed in PBS with 0.2% Triton X-100 for 15 min, followed by one wash step with PBS + 0.2% BSA and a blocking step in 5% normal horse serum (Vector Labs, Burlingame, CA) also for 15 min. Monolayers of HFF cells on coverslips were transfected with Lipofectamine 2000 according to the manufacturer's protocol (Invitrogen). Transfected cells were fixed and permeabilized by the same methods described above. Monoclonal antibodies to ICP4 (Rumbaugh-Goodwin Institute, Plantation, FL), or ppUL44 (gift from Bill Britt, University of Alabama at Birmingham) were incubated with cells for 1 h at 37°C. Monolayers were washed and incubated with a goat anti-mouse secondary antibody conjugated to FITC (Southern Biotechnology Associates, Birmingham, AL). For dual labeling experiments, monoclonal antibodies were directly labeled with a Zenon Texas Red labeling kit as per manufacturer's directions (Molecular Probes, Eugene, OR). After three washes, Vectashield mounting medium containing DAPI (Vector Labs, Burlingame, CA) was added to each slide along with a glass coverslip. Cells were examined with a Nikon TE2000 Microscope using a 40 × objective. Fluorescence images of stained cells were captured with Hamamatsu ORCFA-100 digital camera and recorded using Simple PCI software. All photographs were prepared in Adobe Photoshop CS.

### Immunoprecipitations

T-25 flasks or 6-well dishes of confluent HFF cells, C1-114.373, N3-114.373, or EGFP.373 cells were infected with AD169, Towne, UL114 CTAG, or UL114 NTAG at an MOI of 5 PFU/cell. At 24 and 48 hpi, the monolayers were washed with PBS and lysed on ice in 500 μl of lysis buffer containing 50 mM HEPES, pH 7.5, 150 mM NaCl, 2.5 mM EGTA, 1 mM EDTA, 1% Triton X-100, 1 mM PMSF, and 1 proteinase inhibitor tablet (Boehringer Mannheim). Cell lysates were preadsorbed for 1 h at 4°C with 30 μl of a 50% suspension of protein A-Sepharose in lysis buffer. Proteins were precipitated with 30 μl of the protein A-Sepharose suspension, and 1 μl of a monoclonal antibody to the EGFP domain (Clontech, Palo Alto, CA), the ICP4 epitope tag, or ppUL44 (Rumbaugh-Goodwin Institute, Plantation, FL). The tubes were rocked overnight at 4°C. The protein A-Sepharose beads were washed twice in lysis buffer and once in lysis buffer with the addition of 0.1% SDS, and 1% sodium deoxycholate (RIPA). Samples were boiled 5 min in 80 mM Tris, pH 6.8, 2% SDS, 10% sucrose, and 0.004% bromophenol blue and the proteins were separated by SDS polyacrylamide electrophoresis (48) and transfered to an Immobilon-P membrane (Millipore) at 200 mA for 1 h. Blots were blocked at room temperature for 30 min with 5% (w/v) skim milk in 50 mM Tris, pH7.5, 0.2 M NaCl, 0.01% Tween 20, and incubated at room temperature for 1 h with a monoclonal antibody specific for ppUL44, ICP4, or EGFP diluted 1:1000 in washing buffer (1% (w/v) skim milk, 50 mM Tris, pH7.5, 0.2 M NaCl, 0.01% Tween 20). Blots were washed three times with washing buffer, and incubated at room temperature for 1 h with anti-Mouse IgG(H+L) HRP conjugate (New England Biolabs) diluted 1:2500 in washing buffer. The unbound secondary antibody was removed in three washes with 50 mM Tris, pH7.5, 0.2 M NaCl. The membrane was subsequently developed with LumiGLO or ECL according to the manufacturer's recommendations and used to expose Kodak biomax film.

## List of Abbreviations

HCMV human cytomegalovirus

HIV human immunodeficiency virus

dUTP deoxyuridine triphosphate

RT reverse transcriptase

HEL human embryonic lung

UNG uracil DNA glycosylase

PCNA proliferating cell nuclear antigen

EGFP green fluorescent protein

SCID severe combined immunodeficiency

FITC fluoroscein isothiocyanate

MEM minimal essential medium

TR texas red

DNA deoxyribonucleic acid

## Competing interests

Some of the authors of this publication are supported financially by salary and shares of MedImmune Inc. The authors declare that they have no other competing interest.

## Authors' contributions

MNP generated the recombinant viruses and cell lines described here, performed the complementation experiments and made the yeast mutagenesis constructs. HL performed the immunoprecipitations. GMD and MD worked on the yeast mutagenesis system and assisted in the production of recombinant viruses. CM worked on the immunofluorescence studies. ZW performed the TaqMan analyses. GK and ERK provided critical intellectual input.
